# PEDOT:PSS-Based Piezo-Resistive Sensors Applied to Reinforcement Glass Fibres for *in Situ* Measurement during the Composite Material Weaving Process

**DOI:** 10.3390/s130810749

**Published:** 2013-08-16

**Authors:** Nicolas Trifigny, Fern M. Kelly, Cédric Cochrane, François Boussu, Vladan Koncar, Damien Soulat

**Affiliations:** 1 Université de Lille Nord de France, Cité Scientifique, F-59000 Lille, France; 2 ENSAIT, GEMTEX, F-59100 Roubaix, France; E-Mails: fern.kelly@ensait.fr (F.M.K.); cedric.cochrane@ensait.fr (C.C.); francois.boussu@ensait.fr (F.B.); vladan.koncar@ensait.fr (V.K.); damien.soulat@ensait.fr (D.S.)

**Keywords:** piezo-resistive sensor, fibre sensor, textile composite, 3D warp interlock fabric, *in situ* monitoring, weaving process

## Abstract

The quality of fibrous reinforcements used in composite materials can be monitored during the weaving process. Fibrous sensors previously developed in our laboratory, based on PEDOT:PSS, have been adapted so as to directly measure the mechanical stress on fabrics under static or dynamic conditions. The objective of our research has been to develop new sensor yarns, with the ability to locally detect mechanical stresses all along the warp or weft yarn. This local detection is undertaken inside the weaving loom in real time during the weaving process. Suitable electronic devices have been designed in order to record *in situ* measurements delivered by this new fibrous sensor yarn.

## 1. Introduction

Globally, in all transportation domains, there is a need to reduce the mass of transport vehicles and structures in order to optimize the consumption of energy [1]. This optimization of energy is directly linked to the mass gain and can be solved by the extensive introduction of composite materials as an alternative replacement to steel parts. Moreover, the higher the speed of a vehicle, the lighter the replacement materials must be. Composite materials, developed in the aerospace, aeronautic, automotive, railway and nautical fields for high speed vehicles, are required to be designed to be the best with respect to the technical performance and weight reduction, coupled with the adapted price for the targeted market.

Within the aircraft industry in Europe, new environmental requirements and regulations must be met. These requirements have been created by the European Advisory Council for Aeronautical Research in Europe (ACARE) platform [2] as a result of the Kyoto Protocol's main objectives. In the new generation of aircraft a number of metallic parts, particularly in some critical high dynamic parts such as turbines of jet engines, are replaced by composite materials with the same resistance. This is in order to decrease the overall weight, therefore reducing the fuel consumption. High quality requirements of these composite materials are necessary in order to maintain their performance during their lifetime within the aircraft. Thus, every step of production of the manufacturing processes of composite materials must be monitored to ensure a high quality level. Fibrous reinforcement of composite materials also needs to be checked at each step of their production—from the yarn spinning to the weaving process. Moreover, the quality of the fibrous materials have to be controlled at different levels, from the lower fibre level (microscopic), to the intermediate yarn level (mesoscopic) and also considering the upper structure level (macroscopic) as a woven fabric.

In order to optimise performance (mechanical and lightweight), the project substitutes stacked laminated structures by multidirectional ones based on 3-D warp interlock fabrics. According to existing solutions of 3D woven RTM composite fan blades [3], the best performances are obtained with precise and comprehensive information on those structures from the very beginning of the manufacturing process. It has also been revealed that 3-D structural composites have a higher performance, compared to 2-D laminates [4]. Taking into account the impact studies achieved on 3D woven composites [5–7], the higher performance has been revealed due to their resistance to delamination [8,9]. The 3D angle warp interlock fabric displays high strength and damage resistance as a consequence of the interlaced structure of the warp and weft between the adjacent layers [10].

The final geometry and mechanical performance of the composites also need to be verified with high precision. Therefore, the ability to measure *in situ* local constraints, in real time, on yarns by a fibrous sensor, particularly a piezo-resistive one, is a highly valuable technique for detecting mechanical damages during the weaving process

Piezo-resistive sensors are well known in the field of smart textiles. Elongation sensors within the human-centered field are typically found in medical or paramedical applications, in order to detect and record human motions for rehabilitation or to monitor patient health [11,12], in sport, to learn good gestures [13,14], recognise sign language or monitor movement, or to give tactile sensibility for a robot [15]. Other fields of application which are non-human centered, are in the monitoring of textile structures such used in parachutes canopies [16,17] or structural composites [18].

In general, strain gauges (mechanical sensors) for textiles are based on resistive materials or structures whose electrical resistance changes reversibly according to an applied stress (elongation or pressure) [19]. The term “piezo-resistive sensor” is commonly used. For mechanical resistive sensors, the sensing mechanism is based on at least one of the three phenomena, as follows:

- Change in electrical resistance of textile structures (e.g., stretching or deformation of textile structure). For example: an elongation measurement system made of a stainless steel (non-elastic materials) yarn crocheted chain on elastic fabric. When the stitches are stretched the efficiency of electrical contact between conductive thread increases and then the electrical resistance of device decreases.
- Change in electrical resistance of material not affected by fillers disconnection, whereby only the gauge dimension affects the electrical resistance [20]. In this case the elongation sensor is made of intrinsically conductive polymer (ICP), where macromolecular chains are randomly oriented (isotropic ICP), and conductive polymer composite (CPC) sensors that are filled with large amount of conductive filler.
- Change in electrical resistance of material affected by fillers disconnection. In this case sensors are also named “Electrical Percolation Type Mechanical Sensors” (EPTMS). For these sensors, elongation induces a geometrical change due to the aspect ratio and also the modification of electrical resistivity (ρ) of the materials. Indeed, if the conductive filler content is near, but above, the percolation concentration, the electrical conductivity is very sensitive to volume change of material, due to stretching. A small volume change breaks a lot of conductive path (disconnections) causing a large variation in the overall resistivity of the sensor materials.

Recently, interest has focused on the possibility to develop mechanical sensors from Intrinsically Conducting Polymers (ICP) also called “synthetic metals”. These materials are inherently conducting or semi-conducting in nature due to the presence of a conjugated π electron in their molecular structure. Polypyrrole (PPy), polythiophene (PT), polyaniline (PANI) and poly(3,4-ethylenedioxythiophene) (PEDOT) offer the best compromise between electrical conductivity, stability and processability. Conducting polymers are either made by direct electro- or oxidative-polymerization or alternatively, polymerized and then oxidized chemically or electrochemically. Conductive polymers are applied either as solid compounds, liquid dispersions or solutions. The liquid versions can be easily applied onto a textile substrate (fibre, yarns, textile surface) by coating methods [21]. Aqueous dispersions of poly(3,4-ethylenedioxythiophene)-poly(styrene sulfonate) (PEDOT-PSS) are currently one of most commonly used ICPs. In general, ICP based sensors, which will act as standard piezo-resistive gauges, can easily be produced on textile fabrics through common coating processes. Likewise, ICP-based conductive fibres can be easily obtained by coating. In the case of PANI coated PET [22], electro conductive fibres with good mechanical properties (*i.e.*, mechanical properties of core fibre) and flexibility have been produced, allowing the ability to knit or weave the fibres [23]. Studies of the electromechanical properties of these products show that coated fibres can be used as fibrous sensors [24]. Also, thin coatings of ICPs produce anisotropic materials whose piezo-resistivity is dependent on the macromolecular organisation. Similarly, the behaviour of pure ICP fibres (PANI for instance) is due to the diameter and stretching applied to the fibre during its production [25,26]. Due to this anisotropy, the resistance of pure PANI fibre decreases with applied load (negative gauge factor, k). This fact could be explained by the macromolecular organisation of the PANI backbone, whereby stress increases the drawing of the backbone and quality of lateral contact between chains.

In this paper we harness the piezo-resistive properties of a PEDOT:PSS-based sensor formulation developed by ourselves [27] and apply to E-glass fibre rovings by coating. The formulation consists of a high conductivity solution of PEDOT:PSS (PEDOT:PSS doped with N-methyl-2-pyrrolidinone, NMP) combined with PVA (to improve the mechanical properties of the dried sensor film). Through rigorous testing, the sensor formulation has been refined, leading to the development of a piezo-resistive sensor with the potential to monitor strains and stresses on the yarn, *in situ* during the weaving process.

## 2. Experimental

### 2.1. Materials and Methods

PEDOT:PSS is available in a wide variety of commercial aqueous dispersions. A high conductivity formulation of PEDOT:PSS, containing 2.58 wt% NMP, (Clevios™ CPP105D, [28]) was obtained from H.C. Starck GmbH (Munich, Germany) and used as received. The composition of the blend can be found in Table 1. The density of the solution is 0.90 g/cm^3^ and its solid content is 1.2%. PVA (polyvinyl alcohol Mw 9,000–10,000, 80% hydrolyzed, 1.25 g/mL, [29]), in pellet form, was attained from Sigma Aldrich (St. Louis, MO, USA). PVA solutions were prepared at two mass weight percentages (9 and 27 wt%) by dissolving PVA pellets in deionized water at 90 °C with stirring for 4 h.

The resistivity of pure dry film of PEDOT:PSS:NMP (Clevios™ CPP105D) is about 2 × 10^−4^ Ω·m [30]. The Polymer Data Handbook, gives the resistivity of pure PVA to range between 3.1 and 3.8 × 10^5^ Ω·m [31].

Preparation of PEDOT:PSS:NMP/PVA solutions included combining Clevios™ CPP105D and PVA at different mass ratios (30, 40, 50, 60, 70, 80, 85, 90, 95 wt%) for 24 h with a magnetically driven paddle [32]. Both 9 wt% and 27 wt% aqueous PVA solutions were used. The dry mass weight ratios of CPP105D to PVA were calculated and are given in Table 2. They range from 5.4 to 71.7 wt% and 1.9 to 45.8 wt% for the 9 and 27 wt% PVA solutions respectively (masterbatches A and B).

### 2.2. Resistivity, Mechanical Testing and Electrical Response

Films of sensor coating solutions were prepared by delivering 0.5 cm^3^ of CPP105D/PVA solution corresponding to the various weight percentages of CPP105D, by micropipette to a track of 80 × 10 mm. The electrical resistance of each track was measured with a standard Ohmmeter. The thickness of the tracks was calculated by the combination of the volume of coating used (500 μL), its area (8 × 10^−4^ m^2^), the dry mass ratio and the mass volume of both PVA (1.25 kg/L) and CPP105D (0.90 kg/L). Resistivity (Ω·m) is therefore determined utilizing the following equation:

(1) $$\rho =R\times \frac{s}{l}$$

where ρ (Ω·m) is the resistivity, R (Ω) is the electrical resistance, s (m^2^) is the section of the track and *l* (m) is the length between the silver electrodes (7 mm).

**Figure 1.**
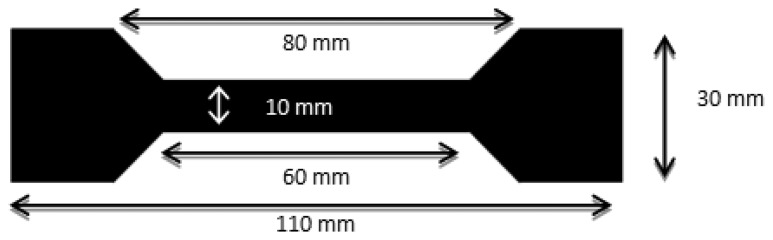
Film dimensions for mechanical testing of CPP105D/PVA films.

The preparation of films for mechanical testing of sensor coatings, included delivering 120 mm^3^ of solutions, containing from 15.1 to 23.7 wt% of dry CPP105D to PVA, into a dog bone shaped mask. The dimensions of the mask are shown in Figure 1.

**Figure 2.**
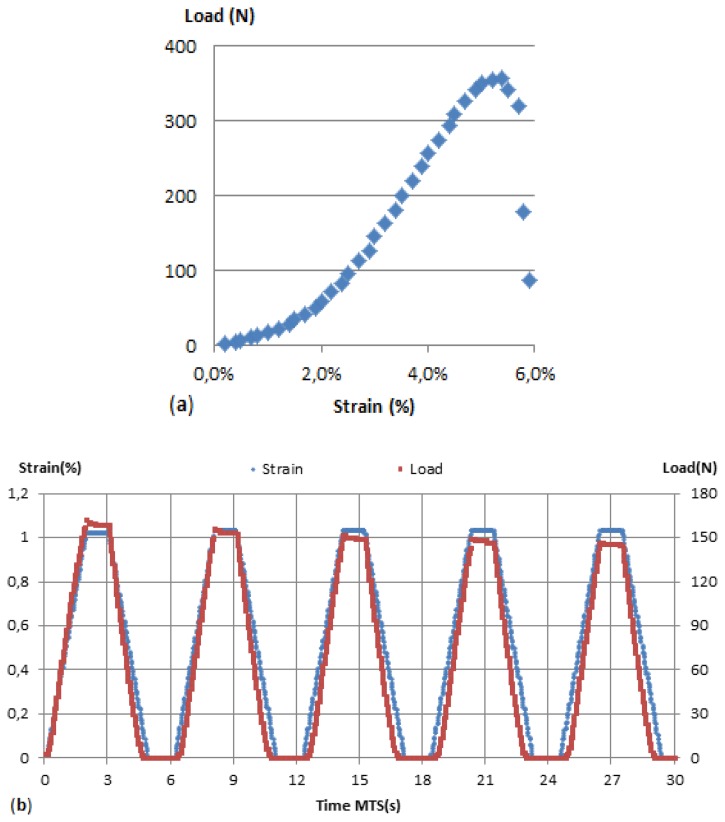
(**a**) Load/strain norm tensile strength test. (**b**) Strain, load *vs.* time multi-cyle tests procedure.

The mechanical properties of the CPP105D/PVA films were investigated using uniaxial tensile tests. The tests were performed using a servo-electric MTS 2/M frame [33,34]. This equipment allowed for precise strains to be applied to the sensors while recording the load response. Characterisation of glass fibres alone and glass fibres with sensor coatings applied were also undertaken on the MTS. The two test procedures utilised were yarn tensile strength at break test (ISO 2062:2009 norm; Figure 2a) and a multi-cycle test (Figure 2b). Both tests have been customised with specific parameters modifications such as speed, elongation, number of cycles or waiting time at both maximum and minimum strain. The standard test parameters were:

- initial distance of the jaws, (initial length, Li): 150 mm
- speed of the jaws: 50 mm/min
- preload: 1.5 N
- maximum strain: 1%
- number of cycle: 5 to 1,000
- waiting time at maximum and minimum strain: 0.25 s to 4 s.

Unless otherwise indicated, these settings were applied for the tests presented in this paper.

In order to measure the electrical response by the sensors, the MTS was linked together with a digital multi-meter (DMM, Keithley 3706) equipped with a fast acquisition card (Keithley 3724). A very high recording speed (In two wire mode, up to 14,000 rec/s) is available. The data acquisition rate was set at different frequencies depending on the tensile tester speed and the duration of the procedure, from 100 Hz to 1,000 Hz.

### 2.3. Design and Production of Yarn Sensors

**Figure 3.**
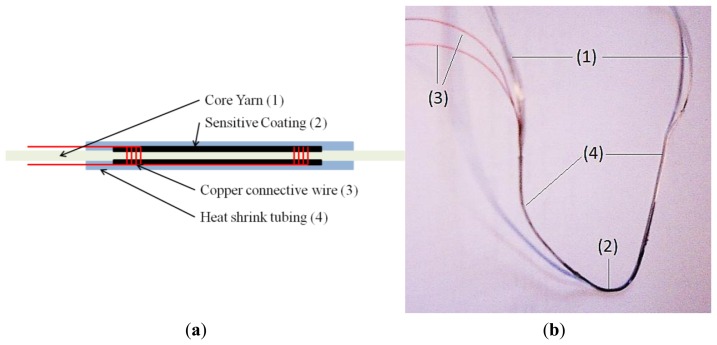
(**a**) Schematic of the sensor yarn; and (**b**) view of the actual sensor yarn, where numbers correspond to those in (a).

Sensors were prepared using glass fibre roving 900 tex (PPG Fibre Glass, Hoogezand, The Netherlands [35]), twisted at 25 t/m as the fibrous yarn substrate. A scheme of the sensor coating applied to the glass fibre is offered in Figure 3a and a view of the sensor yarn in Figure 3b. Before the sensor coating was applied, two polyurethane (PU) insulated copper wires were wrapped around the glass roving. The wrapping zones were spaced 30 or 50 mm apart. The PU insulator was removed before the wrapping. Two successive layers of sensor coatings were applied. Each layer was dried with a hot blow-drying system. When the second layer of coating was dry, a heat shrinking tube (polyolefin) was applied around the sensors to coat and protect it. The total length of the sensor (length of the roving) was approximately 1 m, the length of the connection wire was approximately 2 m and the length of the sensor is 30 to 50 mm (distance between the wrapped connection wires). This last dimension has been defined in function of the final application requiring a short sensing zone in order to be able to measure local stresses in the process, which is operated on a length of approximately 2 m.

## 3. Results and Discussion

### 3.1. Percolation Threshold

**Figure 4.**
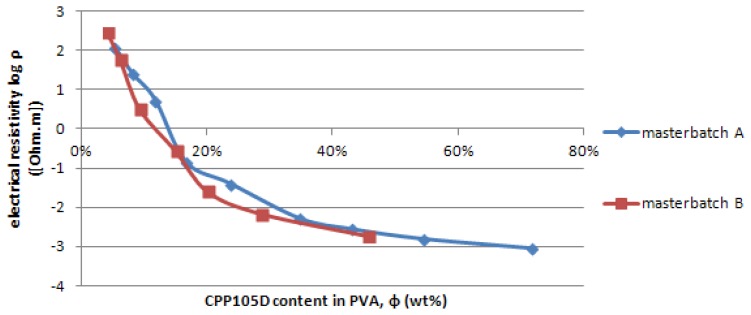
Coating resistivity in function of the dry mass content of CPP105D in PVA.

Resistivity measurements of sensor coatings were undertaken in order to determine the percolation threshold of CPP105D/PVA combinations and consequently to establish the best mass ratio for sensor development, giving the best possible sensitivity. The measured resistivity values for varying dry weight ratios of CPP105D to PVA, are given in Figure 4. The plot from the coating resistivity in function of the dry mass ratio of CPP105D/PVA features only 7 of the 9 different ratios of the 27 wt% PVA blends (Masterbatch B) as “27/30” (1.87 wt% dry CPP105D) and “27/40” (2.88 wt% dry CPP105D) were found to be non-conductive. The resistivity ranges from 115 Ω·m to 9.16 × 10^−4^ Ω·m, and from 281 Ω·m to 1.81 × 10^−3^ Ω·m for CPP105D sensor solutions prepared with 9 and 27 wt% PVA respectively. The resistivity of pure dry film of PEDOT:PSS:NMP (Clevios™ CPP105D) is about 2 × 10^−4^ Ω·m [30]. The Polymer Data Handbook [31], gives the resistivity of pure PVA to range between 3.1 and 3.8 × 105 Ω·m. For both series, 9 and 27 wt% PVA, we observe the same behaviour. From 5.4 to 20.1 wt% of dry CPP105D the resistivity drops by 4 orders of magnitude. Subsequently, the decrease in resistivity is much more mild with a factor of 40 (23.7 wt% to 71.1 wt% mass ratio of CPP105D) for the 9 wt% based blends (Masterbatch A), and a factor of 10 (20.1 wt% to 45.7 wt% dry mass ratio of CPP105D) for the 27 wt% based blends (Masterbatch B).

In this article the percolation threshold has been determined in the middle of the most important slope of the plot representing the coating resistivity in function of the dry mass content shown in Figure 4. There are no exact mathematical methods to determine this percolation threshold particularly in the case where the conductive materials such as CPP105D that are not in the particular form (conductive nanoparticles dispersed in the non conductive matrix) but in aqueous dispersions. However for the applications involving the use of piezoresistive effect for sensors it is probably the most adapted method. The CPP105D is considered well dispersed in the PVA solution.

Therefore, the CPP105D concentration for the coating of sensors must be chosen around 15 wt% in order to benefit from the dramatic change in resistivity to elongation (percolation) and yet be low enough in inner electrical resistance so to not generate noise.

### 3.2. Sensor Calibration

Testing of glass fibre yarns was carried out on the MTS (refer to Section 3.2) in order to characterise their mechanical properties. The glass fibres tend to behave as a quasi visco-elastic material. This mechanical behaviour is especially noticeable on the first test carried out on the yarn. In order to get a more elastic behaviour to stress and to ease the characterization, sensors were “pre-stretched” by a repetition of two to four multi-cycle tensile tests.

**Figure 5.**
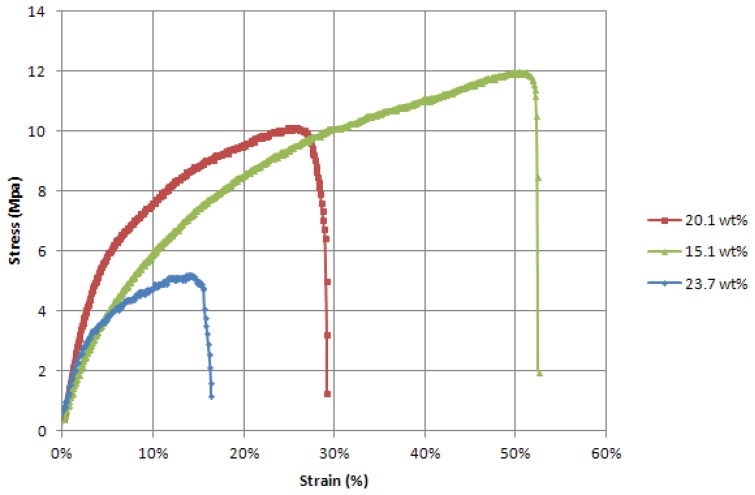
Load/strain curves of the dog bone shaped films of the pure coating at differents dry weight ratios of CPP105D to PVA.

Sensor coating films were prepared and tested for their tensile strength with a breaking test procedure (refer to Section 2.2), so as to record both mechanical behaviour and electrical resistance response to strain. Results from these tests show that CPP105D/PVA films have a solid elastic behaviour. It is worth noting the relatively low ratio of load/strain compared to the glass yarns. In terms of Young's modulus, the coating alone has an average at 150 MPa whereas the 900 Tex glass roving has an average elastic modulus of 5,500 MPa. Strain at breaking is also significantly higher on the coating films (between 15% and 55%) compared to the glass roving (6% at max). As displayed in Figure 5, PVA enhances the mechanical properties of the coating. The breaking strain of the films increases with the wt% of PVA. These properties were expected as they had been previously cited in the work of Chen *et al.* [36].

The electrical resistance of the coating films has also been recorded during tensile breaking tests. The evolution of the resistance is written as a relative variation of resistance compared to the initial resistance R_0_:

(2) $$\frac{\Delta R}{R_{0}}=\frac{R_{i}-R_{0}}{R_{0}}$$

where *R_0_* is the initial resistance of the coating and *R_i_* is the electrical resistance of the coating at the instant of measurement.

**Figure 6.**
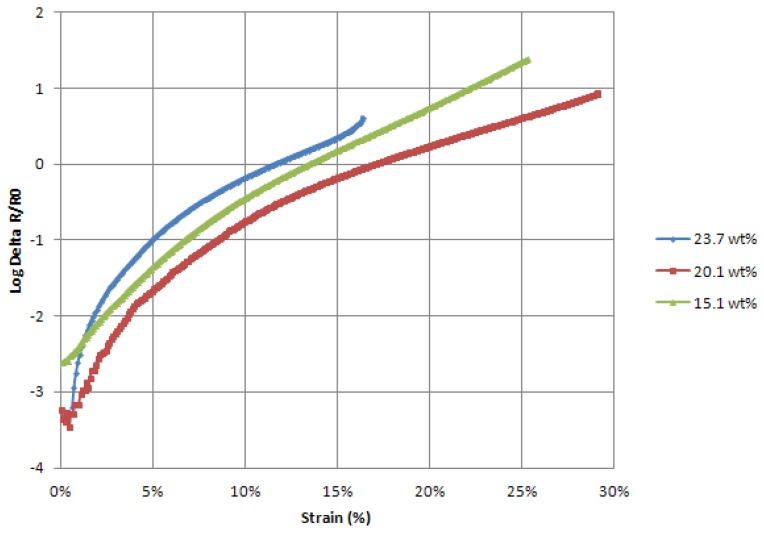
Delta R/R_0_*vs.* Strain of three pure coating films with several ratios of dry CPP105D to PVA.

The evolution of electrical resistance for the films of the pure coating (Figure 6) displays an exponential increase to strain. This observation expresses the change of resistivity of the coating though strain and not only a change of geometry. It is due to our coating mix ratio sitting on the percolation threshold. From this calculation the gauge factor of the film should be computed as a dynamic gauge factor (*k_d_*) which reflects this variation of resistivity through strain, as follows:

(3) $$k_{d}=\frac{d\frac{\Delta \rho }{\rho_{0}}}{dɛ}$$

where ε is the strain and $\frac{\Delta \rho }{\rho_{0}}$ found from the polynomial regression (Equation (4)):

(4) $$\log\frac{\Delta \rho }{\rho_{0}}={\text{a}}_{0}+{\text{a}}_{1}\varepsilon +{\text{a}}_{2}\varepsilon^{2}.$$

The gauge factor varies greatly with the elongation of the sensitive coating. However, for our application, strain variation does not exceed 5% and the typical range of elongation is around 1%. Moreover we tested both films and sensors on quasi-static procedure, at low speed (50 mm/min). Therefore, we made the hypothesis of a static gauge factor (*k*) on a restricted domain of strain (*ε_r_*) (Equation (5)). In our case, the range of gauge factors observed for the films is from *k* = 1 to 3.

(5) $$k=\frac{\frac{\Delta R}{R_{0}}}{{ɛ}_{r}}$$

After individual testing of sensor coatings and glass fibre yarns, fibrous sensors were prepared (Section 2.3) and characterized by multi-cycle quasi-static test procedures (Section 2.2). Data collected via the DMM were locally averaged for every 5 values (if frequency is set at 100 or 250 Hz) or 20 values (1,000 Hz). This delivers a signal, based on the variation of electrical resistance (see Equation (3)), which is thin and clean and fully utilisable:

(6) $$\frac{\Delta R}{R_{0}}=\frac{{\bar{R}}_{20}-R_{0}}{R_{0}}$$

where *R̄*_20_ is the running average on 20 values of R.

The first series of sensors tested featured a 50 mm long coating applied in two consecutive layers directly to the fibres. The dry weight ratios of CPP105D to PVA were 15.1, 16.7, 20.1 and 23.7 wt%.

Our sensors delivered a large disparity in their results. Their expected quality criteria have been defined as follows:

- R_0_: the initial value of electrical resistance of sensors. The lower this value is, the better a sensor it will be, in order to minimize noise during measurements.
- Thinness of signal. A thin, well defined signal is important for analysis.
- Signal stability. The average signal value must remain the same for a same range of strain of the sensor.
- The gauge factor, k. The higher the better as it maximizes the gauge sensitivity. Yet the gauge factor is expected to be between 1 and 3 according to the results of prior tests on thin films.
- For each ratio of dry CPP105D to PVA, some sensors delivered a good signal in terms of thinness and signal stability, yet others do not. From the results obtained it was difficult to identify the proper gauge factor value for each formulation of coating, as some sensors failed to deliver a clean signal with regularity. The initial values of resistance for each sensor, R_0_, remained high. On average the value of R_0_ was 500, 1,000, 300 and 350 kΩ respectively for the 15.1, 16.7, 20.1 and 23.7 wt% dry CPP105D formulations.

### 3.3. Sensor Modification and Improvement

A second series of sensors was developed to minimize these problems discussed above. In order to reduce the R_0_ value, the length of the coating was reduced to 30 mm. Additionally, only the most conductive formulations (20 and 24 wt%) were kept. The goal was to obtain regularity and greater reproducibility from the sensors and therefore a greater predictability. This was achieved by “pre-coating” the section of yarn in between the copper connective wires with pure PVA prior to application of the sensor coating. As a result, the sensitive coating, no longer coats each individual fibre of yarn, but remains only on the outside. By coating only the periphery of the yarn we are able to remove the compression effect noted between the coatings of individual fibres during the strain of the yarn. This compression effect tends to decrease the electrical resistance and counteract the expected behaviour of the sensitive coating.

The yarns “pre-coated” with PVA had three or six consecutive layers of sensor coating applied to measure the influence of this parameter on the regularity of signal delivered and on the performance of the sensors. This series of sensors performed well, displaying clean signals. Although the gauge factors appear very similar for each combination of ratio and number of layers, the R_0_ of the sensors was greatly improved (smaller) by increasing the number of coating layers. Results are summarised in Table 3 below. It is seen that sensor coatings with six layers instead of three, decreased the initial resistivity of them by a factor of 10 on average. The gauge factors of sensors were not influenced by this parameter, neither by the different ratio of CPP105D/PVA, and have an average value slightly over 1. However, this is a very satisfying result, as figures given have been calculated over very large number of tests and cycles for each sensor, proving their consistency.

**Figure 7.**
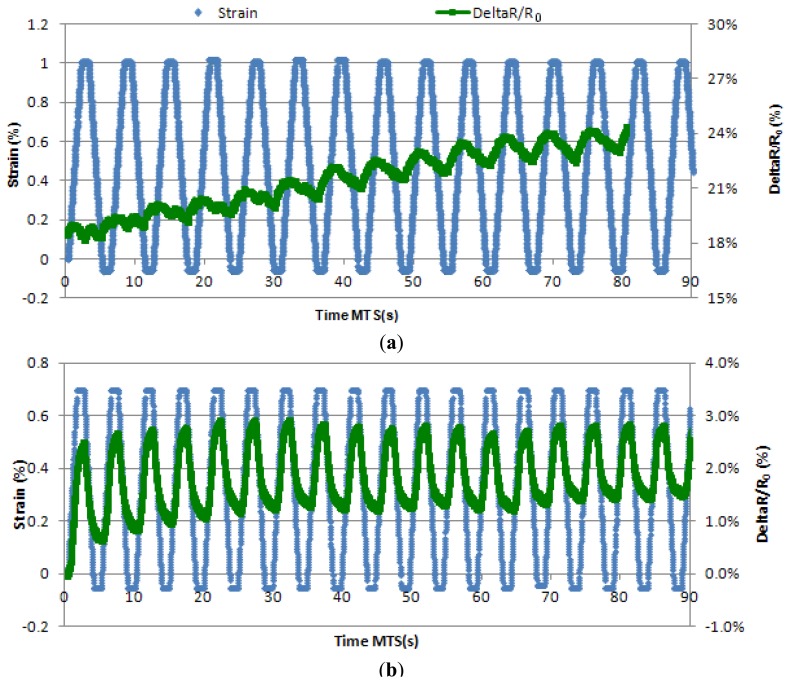
Strain and DeltaR/R_0_*vs.* time between the cycles 0 and 15 of a sensor coated with a 20.1 wt% dry CPP105D formulation. (**a**) is representative from the first series of sensors while (**b**) shows the improvement the second series.

The dramatic improvements observed in Figure 7, led to the characterization of such sensors with a series of long tensile strength test. This type of test has been chosen to replicate “real conditions” and therefore determine the behaviour of the sensors to an extended number of cycles. The “real condition” tests were set at 250 mm/min (5 times faster than the previous ones), with a waiting time of 0.25 s in between strains so that a whole cycle takes approximately 1 s. The number of cycles was increased to 900.

**Figure 8.**
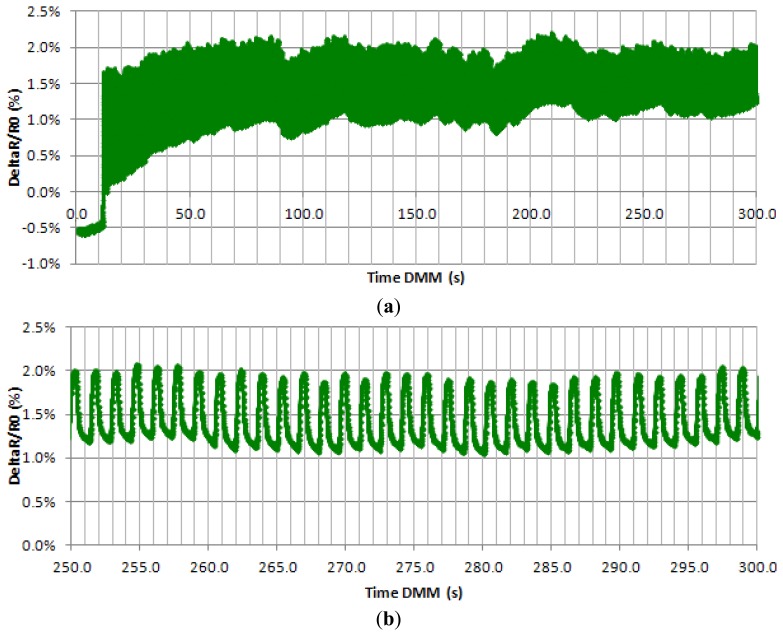
DeltaR/R_0_*vs.* time of a 24 wt% dry CPP105D and six layers coated sensors on a 900 cycles test: (**a**) the first 350 cycles, (**b**) zoom from 250 to 300 s.

In Figure 8 below, we can see a signal given by a sensor attested by “real conditions”. Stability of the signal appears after 60 s of test, when the signal maintains its value between a 1 and 2% variation of resistance (Figure 8a). In Figure 8b, we can also see that the signal is fine, with little noise, and the gauge factor remains around 1 as the strain value at its peak is 0.7%.

## 4. Conclusions

In this work, a new type of sensor capable of recording *in situ* strains and stresses on E-glass fibre yarns, commonly used for yarn sizing application, during their weaving, has been developed. The work has furthered previous studies undertaken at our institute and has been combined with new technologies to meet our specific needs. A new sensitive coating, made from a defined formulation of PEDOT:PSS:NMP (commercially available as Clevios^TM^ CPP105D) and PVA, has been applied on E-glass yarns. Such sensor coating formulations have achieved sound results and show a favourable compatibility with E-glass fibres. However, in order to receive a quality signal, it has been necessary to improve the process of production and adjust its parameters. The reduction of coating length to 30 mm has been undertaken, and an addition of a primary layer of pure PVA on the yarn before the sensitive coating, has been applied. As a result, sensors of the same specification display a consistency in their output signals. Finally, the application of six layers has proved to reduce the initial resistance of sensors to a satisfying average value of 100 kΩ. A successful sensor formulation has been established and is now ready for forthcoming *in situ* measurements within the weaving loom. The 20.1 wt% ratio of dry PEDOT:PSS:NMP to PVA has been selected to be coated in 6 layers after a first “pre-coating” of PVA on a 30 mm length. This new sensor yarn can be reproduced on a large scale while retaining replicable resistivity measurements and sensitivity. The consistency of sensors is important for data acquisition when measuring strains and stresses during the weaving process.

## Figures and Tables

**Table 1.** Clevios™ coating guide formulation CPP 105D.

**Component**	**% By Weight**
Clevios™ P (PEDOT:PSS)	42.92
N-Methyl-2-pyrrolidone	2.58
Silquest^®^ A 187™ (epoxy functional silanes)	0.86
Isopropanol	53.34
Dynol™ 604 (Ethoxylated Acetylenic Diols)	0.30
Total	100.00

**Table 2.** (**a**) and (**b**) Dry mass weight ratios of Clevios™ CPP105D to PVA from masterbatches A and B, respectively.

(**a**)		(**b**)	
	
**Masterbatch A** (9 wt% PVA) wt% liquid CPP105D	**Dry Weight Ratios** (wt% dry CPP105D)	**Masterbatch B** (27 wt% PVA) wt% liquid CPP105D	**Dry Weight Ratios** (wt% dry CPP105D)
30	5.4	30	1.8
40	8.2	40	2.9
50	11.8	50	4.3
60	16.7	60	6.3
70	23.7	70	9.4
80	34.8	80	15.1
85	43.0	85	20.1
90	54.6	90	28.6
95	71.7	95	45.8

**Table 3.** Comparison of the second series of sensors.

**Dry Wt% CPP105D to PVA Ratio**	**20.1**	**23.7**
3 Layers	1.3 < R_0_ < 2.6 × 10^6^ Ω	8 < R_0_ < 10 × 10^6^ Ω
	1.5 < k < 2	k ∼ 1

6 Layers	1.5 < R_0_ < 3 × 10^5^ Ω	3 < R_0_ < 3.5 × 10^5^ Ω
	1 < k < 1.5	1 < k < 1.5
